# Comprehensive Proteomics Analysis of Polyhydroxyalkanoate (PHA) Biology in *Pseudomonas putida* KT2440: The Outer Membrane Lipoprotein OprL is a Newly Identified Phasin

**DOI:** 10.1016/j.mcpro.2024.100765

**Published:** 2024-04-10

**Authors:** Siobhan Kelly, Jia-Lynn Tham, Kate McKeever, Eugene Dillon, David O’Connell, Dimitri Scholz, Jeremy C. Simpson, Kevin O’Connor, Tanja Narancic, Gerard Cagney

**Affiliations:** 1 BiOrbic - Bioeconomy Research Centre, University College Dublin, Belfield, Dublin, Ireland; 2 UCD Conway Institute, University College Dublin, Belfield, Dublin, Ireland; 3 School of Biomolecular and Biomedical Science, University College Dublin, Belfield, Dublin, Ireland; 4 UCD Earth Institute, University College Dublin, Belfield, Dublin, Ireland; 5 UCD School of Biology and Environmental Science, University College Dublin, Belfield, Dublin, Ireland

**Keywords:** bacterial proteomics, bioplastics, carbonosomes, expression proteomics, network biology, PHA granules, polyhydroxyalkanoates, protein interactions, protein networks

## Abstract

*Pseudomonas putida* KT2440 is an important bioplastic-producing industrial microorganism capable of synthesizing the polymeric carbon-rich storage material, polyhydroxyalkanoate (PHA). PHA is sequestered in discrete PHA granules, or carbonosomes, and accumulates under conditions of stress, for example, low levels of available nitrogen. The *pha* locus responsible for PHA metabolism encodes both anabolic and catabolic enzymes, a transcription factor, and carbonosome-localized proteins termed phasins. The functions of phasins are incompletely understood but genetic disruption of their function causes PHA-related phenotypes. To improve our understanding of these proteins, we investigated the PHA pathways of *P.putida* KT2440 using three types of experiments. First, we profiled cells grown in nitrogen-limited and nitrogen-excess media using global expression proteomics, identifying sets of proteins found to coordinately increase or decrease within clustered pathways. Next, we analyzed the protein composition of isolated carbonosomes, identifying two new putative components. We carried out physical interaction screens focused on PHA-related proteins, generating a protein-protein network comprising 434 connected proteins. Finally, we confirmed that the outer membrane protein OprL (the Pal component of the Pal-Tol system) localizes to the carbonosome and shows a PHA-related phenotype and therefore is a novel phasin. The combined datasets represent a valuable overview of the protein components of the PHA system in *P.putida* highlighting the complex nature of regulatory interactions responsive to nutrient stress.

## Highlights

• Comprehensive analysis of *Pseudomonas putida* KT2440 an important industrial microorganism.
• Expression proteomics identified proteins upregulated under PHA-producing conditions. Analysis of the PHA granule mapped a newly identified phasin.
• Protein interaction screen found links between the phasins PhaI and PhaF, and the pyruvate dehydrogenase E1 subunit.

## Graphical Abstract

**Figure:**
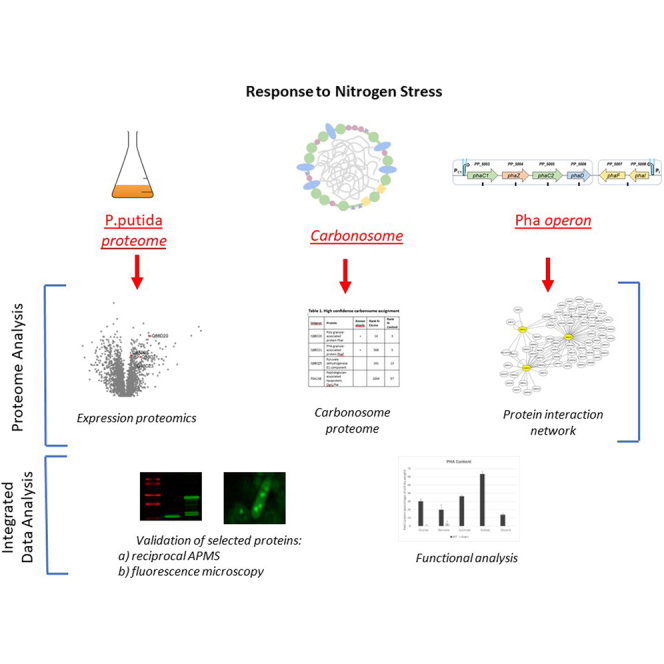

## In Brief

*Pseudomonas putida* KT2440 is capable of producing the biodegradable bioplastic polyhydroxyalkanoate (PHA). To better understand the pathways responsible for PHA metabolism, several types of proteomics experiment were carried out. The proteome response to low nitrogen was examined, followed by cataloguing proteins that physically associate with the PHA storage granule, and a protein interaction network for the key PHA enzyme. The work confirmed the importance of a newly identified phasin (OprL) in PHA biology.

Plastics have become ubiquitous in modern society, being lightweight, durable, and inexpensive. However, approximately 40% of the plastic is produced for single use, with 79% of annual plastic production accumulating in landfills or the natural environment (1, 2). At present, approximately 90% of plastics produced are of petrochemical origin, motivating a search for sustainable biobased alternatives. Polyhydroxyalkanoates (PHAs) are produced by bacteria from renewable sources, are biodegradable, and are of significant interest in the industry, with over 150 different types of PHA on the market (3). This class of polymer therefore has the potential to supply the urgent requirement for novel bioplastics with different physical properties, but for the engineering of efficient bacterial strains to meet manufacturing demands, an improved understanding of their biosynthetic pathways in the context of living cells is needed.

The primary physiological role of PHA in bacteria is carbon storage. Hence, PHA accumulates under conditions of nutrient imbalance. Experimentally, nitrogen limitation is most commonly used to induce PHA production (4, 5, 6), while low levels of other nutrients, including phosphorous, sulfur, or oxygen, also enhance PHA production (7). Early models of PHA biosynthesis emphasized storage in response to nutrient stress, followed by the release of stored PHA during periods of rapid growth. Disruption of the PHA pathway in *P.putida* elicits increased respiration and cell division rates, as well as reduced growth rates (8). However, PHA pathways in different microorganisms have additional roles beyond the regulation of energy and nutrition, for example in resistance to UV radiation (9), protection from osmotic shock (10), and cryoprotection (11). It is becoming clear that PHA metabolism involves continuous synthesis and degradation of the polymer in response to cellular energy and substrate demands that are integrated into the overall cellular metabolic network.

PHA accumulates intracellularly as inclusion bodies, also known as PHA granules or carbonosomes, that are composed of a PHA core surrounded by a layer of proteins, termed granule-associated proteins (GAPs). The exact protein composition of carbonosomes is still under active investigation, but four broad classes have been described: (a) PHA synthases; (b) PHA depolymerases; (c) phasins; and (d) other proteins, for example, acyl-CoA synthase. Phasins are small proteins that localize to the carbonosome, and while they do not appear to have enzymatic activity, they are integral to proper granule formation and segregation. In general, phasins can be considered GAPs for which a granule-related phenotype has been demonstrated (12). Phasins are found in all PHA producers but are not highly conserved across species.

The proteins involved in PHA metabolism in *P.putida* have been well studied individually. Six proteins are encoded within the *pha* locus, comprising *phaC1ZC2D* and *phaFI* operons. *phaC1ZC2D* encodes two polymerases (PhaC1 and PhaC2), a depolymerase (PhaZ), and the Tet-R-like regulatory protein PhaD, while *phaFI* encodes the two phasin proteins PhaF and PhaI. PhaD activates transcription of the *pha* locus in times of carbon excess. Interestingly, PhaD physically interacts with the phasin PhaF, although unlike PhaF it is not a GAP (13). PhaF was originally described as a nucleoid-associated protein, with a disruption phenotype linked to granule localization and segregation during cell division (14). In the same recent paper describing an interaction with PhaD, PhaF was shown to form a complex with PhaI, the other confirmed phasin in *P.putida* (13).

While PhaD most directly regulates transcription from the *pha* locus, other proteins are implicated in the regulation of the broader PHA pathway in *P.putida*. These include global regulators such as RpoN, RpoS, RelA, and Crc that respond to general nutrient excess; the GacS/GacT two-component system; and the PsrA and PTS system that appears to respond to more specific pathways such as fatty acid metabolism. In addition, PhaF is itself capable of negative regulation of phaC1 (14), perhaps *via* the physical interaction with PhaD (13). These observations indicate that a wide network of protein interactions is likely to regulate and mediate PHA metabolism in *P.putida* beyond the immediate steps catalyzed by enzymes encoded by the *pha* locus. Here, we take advantage of recently developed powerful mass spectrometry approaches to investigate these pathways. We first used global expression proteomics to map the response to low nitrogen availability (which stimulates PHA synthesis), next, we focused on the composition of the carbonosomes themselves, and finally, we constructed a protein–protein interaction map of the main players in the PHA biology of *P.putida*.

## Experimental Procedures

### Growth of Bacterial Cells

*P.putida* cells were grown in LB broth at 30 °C, with shaking (250 rpm). 1 ml of overnight culture was inoculated into 50 ml of M9 minimal media with glucose as the carbon source. For PHA accumulating conditions, nitrogen () was limited to 0.1 g/lL; otherwise, nitrogen concentration was 6 g/l. Upon harvest, pelleted bacteria were washed twice with PBS and frozen at −80 °C until further analysis.

### PHA Quantification

After 48 h growth in liquid culture, cells were harvested by centrifugation at 5000*g* for 10 min at 4 °C. Cell pellets were washed with 5 ml PBS and lyophilized using a Labconco (Fischer Scientific) freeze-dryer. Cell dry weights are determined by weighing lyophilized cells. PHA content was determined by subjecting 5 to 15 mg lyophilized cells to acidic methanolysis (15). The 3-hydroxy alkanoic acid methyl esters were analyzed by gas chromatography using an Agilent 6890N chromatograph equipped with an HP-Innowax capillary column and a flame ionization detector. The following temperature program was employed: 120 °C for 5 min, increase by 3 °C/min to 180 °C, 180 °C for 10 min. Total PHA content was determined as a percentage of CDW.

### Nile Red Staining

Nile red stain was carried out on 96-well black plates, according to a modified version of the protocol by (16). Briefly, an 80 μg/ml solution of Nile red in DMSO was prepared. 1 ml of *P.putida* culture was equalized to OD600 nm 0.5. Following centrifugation, the pellet was then resuspended in 1 ml PBS. This procedure was carried out twice, and the pellet was resuspended in 0.5 ml 35% ethanol solution, shaken gently at room temperature for 15 min. The tube was then centrifuged and the supernatant was discarded. 940 ml of PBS was added to the pellet and 70 μl of the Nile red Solution. The pellet was resuspended and rocked in the dark for 4 h. Samples were diluted 10-fold in PBS and 200 μl of the diluted solution was placed in a 96-well black plate. The plate was then read using an excitation wavelength set to 535 nm and emission at 605 nm.

### Isolation of PHA Granules/carbonosomes

Cells were harvested by spinning down at 4 °C at 5,000 rpm for 30 min. They were washed twice with 50 mM PBS and the supernatant was discarded. Cells were lysed using Bugbuster Master Mix (5 ml per 1 g cell wet weight, with a cOmplete Mini, EDTA-free tablet (Sigma). The tubes were incubated in a warm room (37 °C) for 30 min, with shaking. Cell lysate was then added to a polyallomer centrifuge tube containing a glycerol gradient with 10 ml 90% glycerol; 10 ml 50% glycerol. The tube was then filled to capacity with 100 mM Tris-HCl (pH 8). This was centrifuged at 20,000 rpm at 4 °C for 40 min using 32 Ti swinging rotor. The layer was collected at the interface between 90% and 50% glycerol using a Pasteur pipette. The layer was diluted using 100 mM Trizma (pH 8) to a total volume of 10 ml. The diluted sample was then loaded onto a second glycerol gradient. This gradient contained four layers: 90%, 80%, 60%, and 40% glycerol, each 10 ml. This was centrifuged at 20,000 rpm 4 °C for 40 min using 32 Ti swinging rotor. PHA granules settled at the interface between 80% and 60% glycerol. The PHA layer could be identified due to its thick, viscous appearance. This layer was collected and transferred to a fresh polyallomer tube. The collected layer was again diluted 20-fold with 100 mM Trizma pH eight and centrifuged at 20,000 rpm 4 °C for 30 min using 32 Ti swinging rotor. The supernatant was discarded, and the pellets were resuspended in 500 μl 100 mM Trizma pH 8. Granules were stored at 4 °C until further analysis.

### Affinity Purification

Cultures expressing eYFP constructs were harvested after 24 h, pelleted, and lysed on ice using a protein extraction reagent, Bugbuster, supplemented with cOmplete Mini protease inhibitors (Sigma), 30 min, with pipetting every 10 min. The cell lysate was centrifuged at 17,000*g* for 10 min at 4 °C. 300 μl of supernatant was transferred into a fresh precooled tube and 300 μl of dilution buffer was added (10 mM Tris pH 8, 150 mM NaCl, protease inhibitors). 25 μl of GFP-Trap Agarose bead slurry (Thermo) was added to the lysate and gently rotated for 1 h at 4 °C and then centrifuged at 2,500rcf for 2 min. The beads were washed three times, and 25 μl of digestion buffer I (50 mM TrisHCl pH 7.5, 2M Urea, 5 μg/ml Trypsin Singles, 5 mM DTT) was added, before being incubated in a thermomixer at 30 °C, 400 rpm for 30 min. The supernatant was transferred to a fresh microtube and resuspended in 50 μl digestion buffer II (50 mM Tris HCl, pH7.5, 2M Urea, 5 mM IAA). The digest was then incubated overnight at 400 rpm, 32 °C. The reaction was stopped by adding 3 μl of formic acid. The peptides were desalted, adsorbed onto C18 zip tips, eluted in high acetonitrile, and separated online by nano-chromatography interfaced with a Q Exactive mass spectrometer.

### In-Solution Trypsin Digest and Mass Spectrometry

Frozen bacterial pellets were thawed on ice and resuspended in 8M urea. Proteins were treated with trypsin as described (17). Peptide samples were introduced Q Exactive mass spectrometer *via* an EASY-nLC 1000 UHPLC system (Thermo Fisher) coupled to an in-house packed C18 column (New Objective). Parent ion spectra (MS1) were measured at resolution 70,000, AGC target 3e6. Tandem mass spectra (MS2; up to 10 scans per duty cycle) were obtained at resolution 17,500, AGC target 5e4, and collision energy of 25. Data were processed using MaxQuant (v1.6.5.0) (18) using the *P.putida* KT2440 FASTA protein database, downloaded from Uniprot on 03.01.2019 (5527 entries). The following search parameters were used: Fixed Mod: carbamidomethylation; Variable Mods: methionine oxidation; Trypsin/P digest enzyme; one missed cleavage; Precursor mass tolerances 6 ppm; Fragment ion mass tolerances 20 ppm; Peptide FDR

One%; Protein FDR 1%. Potential contaminants, proteins identified using reverse sequence databases, and those identified based on modified peptides, were removed. Statistical tests and volcano plots were performed on Perseus (v1.5.5.3) (19).

### Confocal Microscopy

Cells expressing eYFP-tagged proteins were grown in nitrogen-limited media, as detailed earlier, for 24 h 1 ml of culture was centrifuged, washed in PBS, and fixed using 10% neutral-buffered formalin (NBF) for 10 min. The cultures were stained by mixing with 0.1 volume of Nile red (0.1 μg/ml in acetone). 10 μl of stained, fixed culture was applied to a microscope slide and 10 μl of Mowiol mounting medium (2.4 g Mowiol 4–88 (Millipore); 6g glycerol in 6 ml of water). A cover slide was added and the slides were left overnight. Next, 12 ml of 0.2 M Tris-Cl (pH 8.5) was added, the slides were heated to 50 °C for 10 min, The solution was clarified by aliquoting 1 ml into Eppendorf tubes and centrifuging for 15 min at 10,000 rpm. PHA granules and eYFP-fused proteins were visualized using a confocal laser scanning microscope, Zeiss LSM 800 Airy microscope. Images were acquired using an 63X objective in Airyscan mode. For eYFP detection, the wavelength was set to 490 to 550 nm; for Nile red 575 to 7000 nm.

### Engineering of *oprL* Null Mutant

*oprL* was scarlessly deleted using a λRed/Cas9 recombineering method as described (20).

### Data Analysis

Network display of protein-protein interactions was performed using Cytoscape (v3.1.1) (21). Pathway analysis was carried out on ClueGo, a plugin of Cytoscape (22). Software platforms used to predict protein biophysical factors included: IUPred2A (estimate the probability of disordered regions in proteins of interest) (23); SignalIP50 (predict signal sequences) (24); TMHMM2.0 (search for predicted transmembrane domains) (25).

### Experimental Design and Statistical Rationale

Three technical replicates were employed for all proteomics experiments (expression proteomics, carbonosome purification, protein interaction screens), as well as for bacterial growth curve and PHA analysis experiments. The Student’s *t* test was used to evaluate significance in pairwise comparison experiments.

## Results

**Fig. 1:**
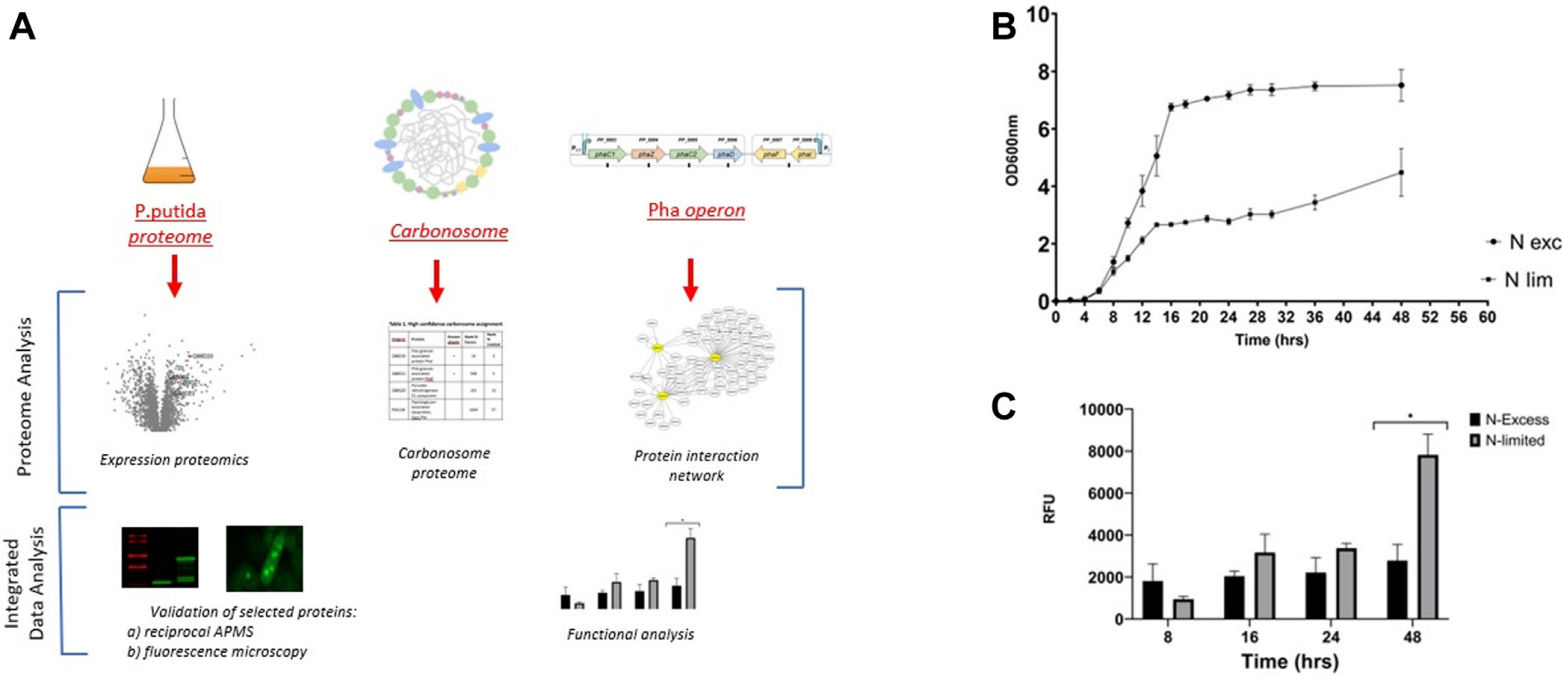
**Comprehensive proteomics analysis of the PHA pathway in *Pseudomonas putida*.***A*, overview of the study. *B*, growth in nitrogen-limited (N-lim) and nitrogen excess (N-exc) conditions, using glucose as a carbon source. *C*, PHA levels were monitored using Nile red stain (∗paired *t* test, *p* < 0.05).

We designed a comprehensive series of experiments to investigate three aspects of protein level regulation of the PHA pathway in *P.putida*: (i) the global proteome response to nitrogen stress; (ii) the protein complement of isolated carbonosomes and (iii) a protein interaction network focused on the *phaC1ZC2D* and *phaFI* regulons (Fig. 1*A*). We used reduced nitrogen limitation in a growth medium containing a glucose source of carbon to stimulate PHA production. Cells grown in reduced nitrogen showed a reduced growth rate (Fig. 1*B*), while Nile red staining confirmed that PHA production was indeed stimulated by low nitrogen levels, particularly after 24h (Fig. 1*C*).

### Global Proteomics of *P.putida* in Nitrogen-limiting Media

**Fig. 2:**
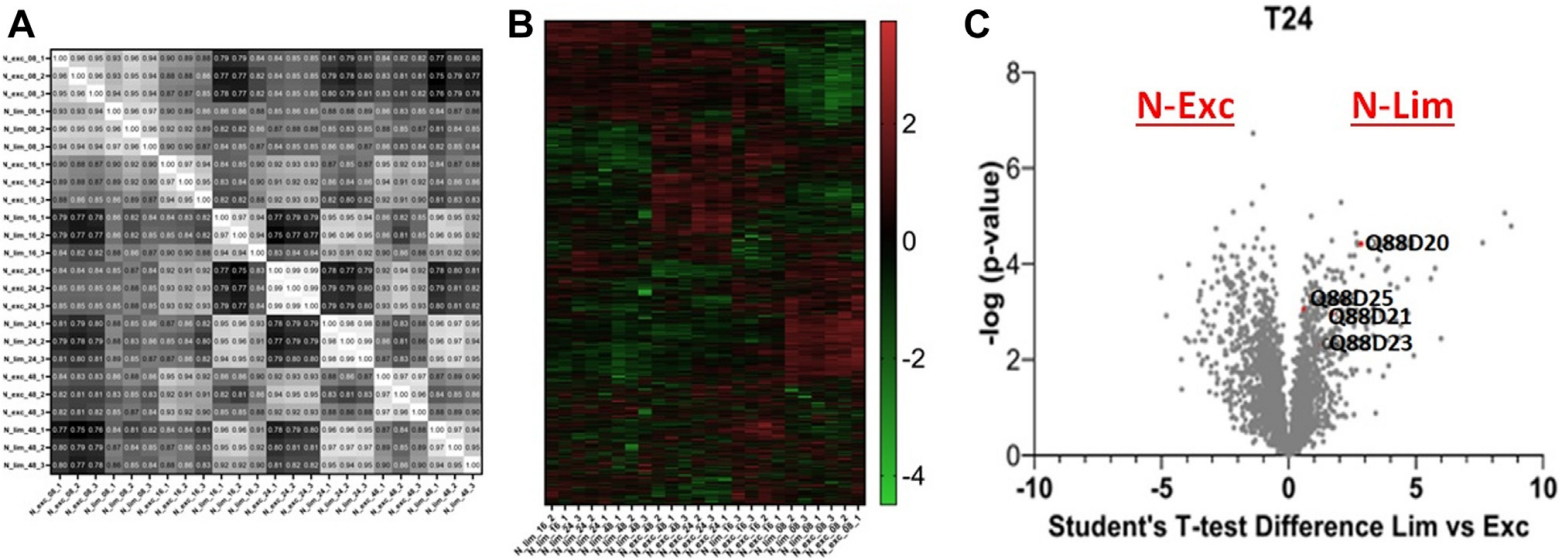
**Expression proteomics of the response to nitrogen limitation in *P.putida*.***A*, correlation comparison of expression proteomics readouts for all experiment pairs. Cells were grown in nitrogen-excess (N_Exc) or nitrogen-limited (N_Lim) conditions, and harvested at the indicated time points. Suffixes (−1,-2,-3) indicate replicate experiments (n = 3). *B*, heat map of protein expression levels following growth in N_Lim and N_Exc conditions using glucose as the carbon source. *C*, *Volcano plot* comparing the relative expression of individual proteins from cells grown in N-Lim or N-Exc conditions (paired *t* test).

To improve our understanding of how PHA production is controlled when *P.putida* accumulates PHA, we compared growth in nitrogen-limited (PHA accumulating) and nitrogen-excess (PHA non-accumulating) conditions. Samples were taken at 8, 16, 24 and 48 h post-inoculation, to reflect the exponential, early stationary, mid stationary, and late stationary phases of *P.putida* growth, respectively. Overall, 1764 proteins were identified and quantified (relative abundance determined using label-free proteomics) across 24 samples (n = 3 for each time point), representing approximately 33% of the theoretical *P.putida* proteome (Supplemental Table S1). This is comparable to similar bacterial proteomics experiments, for example in *Escherichia coli* (26). Pairwise comparison of the proteome readouts confirmed that technical replicates are highly reproducible (Spearman’s Rank Correlation Coefficient ≥0.94), giving confidence that the proteomics platform can sensitively identify protein expression changes responsive to experiment (Fig. 2*A*).

The overall experiments can be visualized using a heat map of protein expression levels subjected to 2D-hierarchical clustering (Fig. 2*B*). Broad patterns of co-regulated proteins are apparent, and interestingly, the N-limited and N-excess samples tend to co-cluster except for the early 8 h time point. This suggests that proteome-level changes in response to nitrogen availability are largely established by the stationary phase. We, therefore, focused on expression changes for the t = 24 h samples in subsequent experiments (Supplemental Table S2; Fig. 2*C*). 169 proteins were found to be upregulated in N-limited culture (including the phasins PhaI and PhaF, and PHA polymerase PhaC2), and 267 in N-excess culture. These observations agree with earlier omics studies, including a combined proteomics/transcriptomics/metabolomics analysis of single and combined depletion of carbon and nitrogen sources from growth media during culture of *P.putida* KT2440 (27), as well as an analysis of the proteome of *P.putida* KT2440 grown under phosphorous-carbon limitation conditions in continuous culture (28). Furthermore, the up-regulation of PhaI, PhaF, and PhaC2 also agrees with an expression proteomics analysis of the *P.putida* CA-3 strain (6). Enrichment analysis (for Gene Ontology cellular pathways; Interpro for protein structural domains) of these sets of up- and downregulated proteins found significant overexpression of ABC transporter family members, perhaps reflecting attempts at nutrient uptake from the environment (Supplemental Table S3). Proteins involved in nitrogen metabolism were also found to be upregulated in nitrogen-limited growth media, for example, the key nitrogen-responsive transcription factor NtrC (Q88CY1), and glutamine synthetase (Q88CY3). Overall, our protein expression analysis generated a database of proteome-wide changes, identified individual proteins whose expression altered in response to nitrogen stress, and confirmed expected changes focused on the PHA and related pathways.

### Proteomics of the *P.putida* Carbonosome

We next attempted to describe the protein complement of the carbonosome. While the biology of the major phasins has been studied for many years, attempts to identify the PHA granule proteome vary widely in reported numbers of proteins (10s to 100s), depending on the organism used and on the sensitivity of the method used. For example, Catone and coworkers (29) reported 10 proteins identified from PHA granules of *Pseudomonas extremaustralis*, while (30) identified 15 in *Herbaspirillum seropedicae*. In contrast (31), identified over 400 proteins in carbonosomes from *Ralstonia eutropha*, while (13) identified a similar number in *P.putida*. SDS-PAGE analysis of the *P.putida* carbonosome proteome shows that approximately 5 to 10 proteins dominate by mass abundance, suggesting that while many of the large numbers of low abundance may be *bone fide* and even functionally relevant carbonosome proteins, many may arise from carryover during biochemical purification steps. We therefore sought to identify carbonosome-associated proteins using the sensitive gel-separation: LCMS technology and combine this with a stringent bioinformatic workflow to evaluate whether a protein was likely granule-associated or a background contaminant (Supplemental Table S4).

The data analysis approach we employed had two main stages: enrichment- and knowledge-based. In the enrichment stage; we asked if proteins were enriched in the granule fraction relative to the whole proteome (89 proteins >2-fold enriched), and also if the proteins were enriched in N-limited conditions relative to N-excess conditions (100 proteins >2-fold enriched). In other words, we imposed a requirement that potential GAPs were observed to be upregulated under conditions known to promote PHA granule biosynthesis. In the knowledge-based approach, we used calculated and predicted biophysical properties, as well as information from published data, to help assess the likelihood of an enriched protein being a *bone fide* GAP. Since GAPs are highly diverse, it was not possible to exclude any protein based on one criterion alone, so this information was collected as a database and used to assemble a list of high-confidence potential GAPs (Supplemental Table S4).

We also compared the proteins reported recently in a study of isolated *P.putida* carbonosomes by Tarazona and coworkers [2020] using MALDI mass spectrometry, to proteins we detected in the PHA granule fraction. Of the 78 proteins identified by Tarazona, 75 were found in our granule isolation experiment, suggesting that our glycerol purification/LCMS approach is comprehensive. Other useful data that can help discriminate true granule proteins from false positives include properties that have been observed in some (but not all) confirmed GAPs, including the presence of disordered regions, signal peptides, and transmembrane domains. Using hydrodynamic, spectroscopical, and thermodynamic experiments, the *P.putida* phasin PhaF has been shown to have a partially disordered N-terminal domain in the absence of PHA (32). Since some phasins have predicted disordered regions, we used PONDR software to predict regions of disorder in potential GAPs. Another disorder prediction software, ESpritz was used to predict the overall percentage of disorder in each protein detected in the granule fraction. Proteins containing a signal sequence are likely to be secreted and/or retained in the cell membrane. The presence of these features was predicted using SIGNALP5.0 for signal peptides. The TMHMM2.0 program was used to search for protein regions predicted to contain transmembrane domains. Finally, the two *P.putida* phasins PhaI and PhaF contain known phasin domains (designated IPR008769 by the Interpro database and PF05597 by the Pfam protein family databases). The entire *P.putida* proteome was searched for the presence of these domains. Although this domain is present in over 263 sequences across 196 species (12), PhaI and PhaF are the only proteins in the *P.putida* KT2440 proteome with this domain. Other proteins in *P.putida* have been shown to be granule-associated and do not contain this domain, for example, PhaZ and acyl-CoA synthase. Hence, although the presence of a Phasin domain is indicative of a protein being associated with the carbonosome, its absence does not exclude a protein from being a GAP.

Based on these analyses, four proteins—PhaI, PhaF, the outer membrane lipoprotein OprL, and the E1 component of the pyruvate dehydrogenase complex (PDH-E1)—were selected as high-confidence GAPs (Table 1). In our study, they were enriched in both the carbonosome proteome (ranked first, third, 10th, and 25th by abundance) and the N-limited proteome (ranked third, fifth, 57th and 13th most upregulated). Furthermore, both PhaI and PhaF were identified in the Tarazona study. OprL and PDH-E1 were not identified in the carbonosome preparation of Tarazona; however, the porin OprF (Q88L46), which shares 35% amino acid homology with OprL, was identified. Other proteins shared several of these properties and overlapped with the proteins identified in the carbonosome proteome analysis by Tarazona, including the 30S ribosomal protein L3 (Q88QN5), the OmpA family protein (Q88NT3), and the peptidoglycan-associated lipoprotein (Q88PU5). The former was considered not to be localized to the carbonosome by Tarazona and coworkers based on additional microscopy evidence. While Q88NT3 and Q88PU5 were found in all our replicate carbonosome proteome experiments, they were present at low levels (0.7% and 0.6% of total MS ion signal respectively) relative to the most abundant carbonosome protein PhaI (3.8%). Therefore, while they may potentially be true components of the carbonosome, we excluded them from further analysis since they cannot be considered high confidence.

**Table 1:** High confidence carbonsome assignments

Uniprot	Protein	Known phasin	Rank N-Excess	Rank N-Limited	Tarazona^a^	Granule Enriched^b^	N-Lim Enriched^c^	TM domain^d^	Signal P5.0^e^	% disorder^f^
Q88D20	Poly granule-associated protein PhaI	+	14	3	+	+	+	0	0.01	23.74
Q88D21	PHA granule-associated protein PhaF	+	568	5	+	+	+	0	0.01	85.44
Q88QZ5	Pyruvate dehydrogenase E1 component		161	13		+	+	0	0	6.47
P0A138	Peptidoglycan-associated lipoprotein, OprL/Pal		1004	57	+		+	0	0	53.01

a Identified as a potential GAP by (13).

b > 2-fold enriched in carbonosome relative to global proteome (LFQ, Label-free quantitation).

c > 2-fold enriched in N-limited proteome relative to N-excess proteome (LFQ, Label-free quantitation).

d Transmembrane domain predictions TMHMM2.0.

e Signal peptide predictions SignalP5.0 (24).

f Disorder predictions PONDR (50).

### PHA-Centered Protein Network: Round one

Mapping the physical interactions of a protein can help in understanding the biology of that protein by placing it in the context of the wider cellular network of interacting proteins and pathways. In particular, we aimed to gain insight into how PHA production is linked to the metabolic architecture of a *P.putida* cell. While there have been functional studies on individual PHA-related proteins, few protein interaction screens have been attempted for this species. Here, we carried out a two-round physical interaction study, the first focused on proteins directly encoded in the *pha* locus, the second to confirm any findings, and to investigate proteins identified in the first round.

**Fig. 3:**
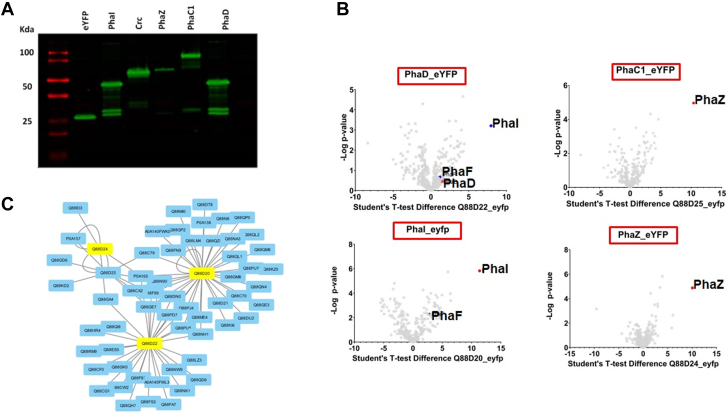
**PHA-centered protein-protein interaction screen.***A*, expression of bait-eYFP fusion proteins. Cells transformed with relevant plasmids were harvested after 24 h growth in nitrogen-limited media, separated by SDS-PAGE, and analyzed for fluorescent signal. *B*, *Volcano plots* comparing the relative levels between bait-specific and null control APMS experiments. *C*, cytoscape network representation of putative physical protein-protein interactions identified in the screens.

For Round 1, since no suitable antibodies were available for affinity purification of PHA pathway proteins, we implemented a fusion protein strategy, fusing enhanced yellow fluorescent protein (eYFP) to the C-terminal of PhaI (Q88D20), PhaD (Q88D22), PhaZ (Q88D24), and PhaC1 (Q88D25). To confirm that the fusion proteins were correctly expressed, samples were analyzed by Western blot using an anti-eYFP antibody, with each fusion protein co-migrating with markers of the expected size (Fig. 3*A*). Cultures expressing the fusion proteins were grown on nitrogen-limited media, harvested after 24 h, subjected to affinity purification mass spectrometry (APMS) using anti-eYFP antibodies and on-bead trypsin digestion, and analyzed by LCMS to identify potential interactors. For each bait protein, volcano plots were generated projecting the *t* test difference recorded for each protein against *p*-value (Fig. 3*B*). By comparing the MS signal for each protein from prey pulldowns with null control (nonfusion eYFP) pulldowns, potential interactions were mapped. Despite the use of statistics, protein pairs identified in screens like this are best considered potential interactions until confirmed by an independent experiment. Overall, our data largely recapitulate interactions expected based on earlier reports, or on the known biology of PHA in *P.putida*. However, new putative interactions were identified (discussed below). The Cytoscape platform was used to generate a protein interaction map using the data compiled from all potential interactors identified in the primary screen, for the three PHA operon proteins studied: PhaI, PhaC1, and PhaZ (Fig. 3*C*). The network structure suggests that each protein operates within somewhat discrete subnetworks. Most of the shared interactions are with lipoproteins or ribosomal proteins, which may be artefactual. Interestingly, only two putative interactions between PHA pathway components were observed: PhaI →PhaF and PhaI → PhaD. This suggests that although PhaI, PhaC, and PhaZ are all located on the PHA granule and involved in PHA metabolism, they may not form mutual physical interactions (Supplemental Table S5).

Thirty-five proteins (including the bait) were identified as potential phasin PhaI binders. These included six ribosomal proteins and two subunits of RNA polymerase, which may be artefactual. The phasin PhaF was among the potential PhaI interactions in this experiment, in agreement with recent work (13). Twenty-nine proteins (including the bait) were identified as potential interactors with transcription factor PhaD, including six lipoproteins, and a variety of metabolic enzymes. These cover a range of cellular functions of no obvious relation to PHA. Four proteins (including the bait) were identified as potential binders of PHA depolymerase PhaZ. Nine proteins (including the bait) were found to be significantly enriched in the PHA polymerase PhaC1 APMS experiment. Five are classed as putative lipoproteins, none of which were observed in our carbonosome proteome (although one, Q88N90 was found in a carbonosome purification by Tarazona and coworkers [2020]). Q88N90 and Q88F99 were also found as potential interactors with PhaI and PhaD, and they were upregulated following growth in N-limited media (Supplemental Table S2). However, other authors have argued that the presence of lipoproteins on the PHA granule may be artefactual, arising from the purification steps (31). We therefore decided to carry out additional experiments on one representative lipoprotein, Q88F99.

Two proteins of particular interest were identified in these screens. Pyruvate dehydrogenase E1 component (Q88QZ5) (PDH-E1) was found to be a potential interaction with PhaI. Although not found by Tarazona in their carbonosome preparation, we observed PDEH1 to be upregulated in cells grown on N-limited media and in our carbonosome preparation. Given its potential connection to central metabolism, we selected PDH-E1 for further study. The outer membrane lipoprotein OprL (P0A138) was also highly enriched in this APMS experiment, as well as being found in the carbonosome purification of (13). OprL is also known as Pal, the outer membrane component of the Pal-Tor system, which functions with Tor proteins associated with the inner membrane in cell wall biogenesis. We did not detect Tol proteins in this experiment. Given these observations, we selected OprL for additional experiments.

### PHA-Centered Protein Network: Round two

**Fig. 4:**
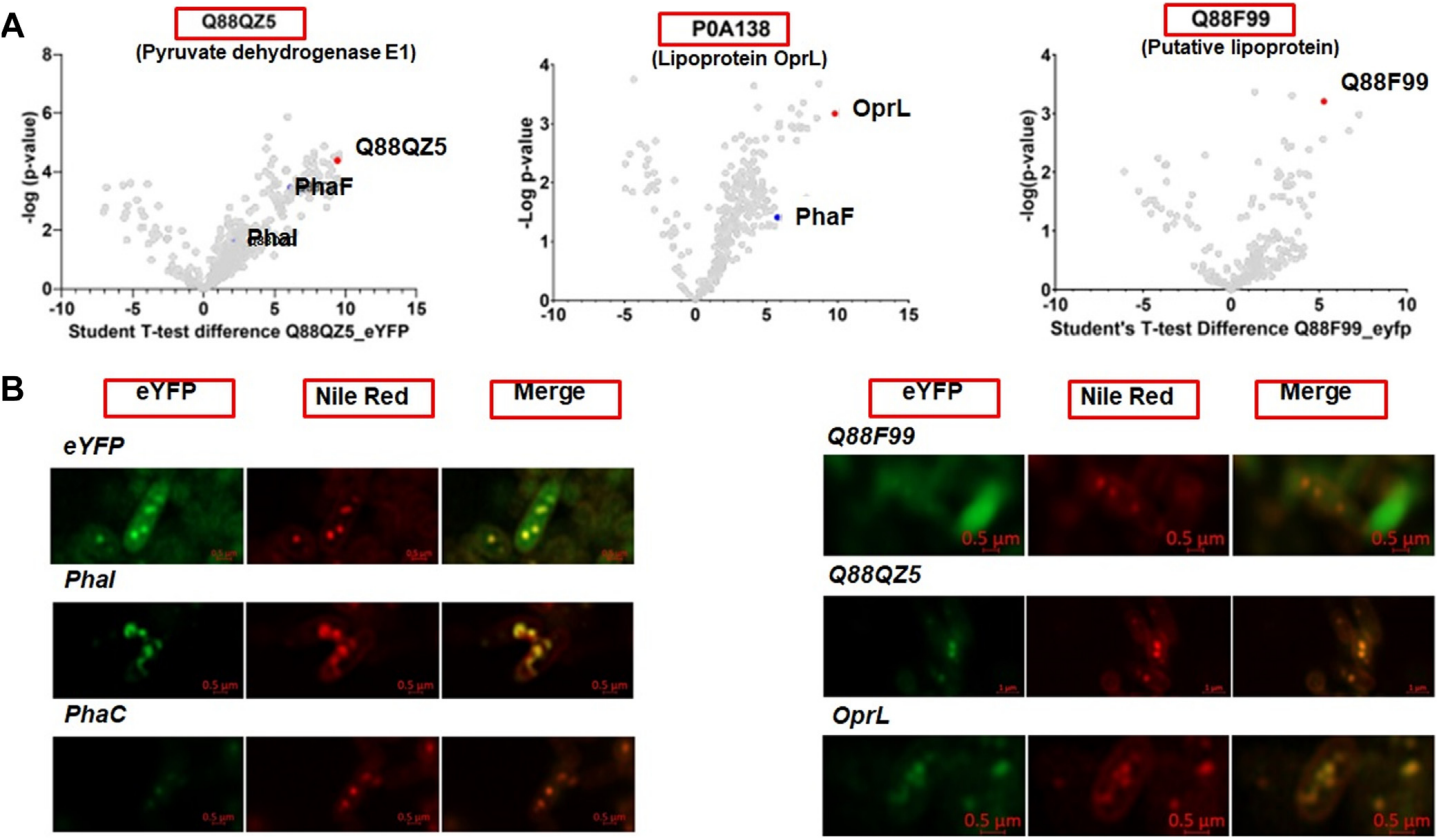
**Validation of protein-protein interactions.***A*, *Volcano plots* comparing the relative levels between bait-specific and null control APMS experiments. *B*, fluorescence microscopy and Nile red staining of *P.putida* strains expressing the fusion proteins indicated.

Since several proteins not previously implicated in PHA biology were identified as potential interactors in multiple PHA-related protein pulldowns, a second, independent protein interaction screen was implemented to validate primary findings and further investigate their biology. As mentioned, we chose PDH-E1, putative lipoprotein Q88F99, and OprL as baits for the second round of APMS experiments. Again, the lack of suitable antibody reagents precluded validation by co-immunoprecipitation. The secondary screen was therefore based on the premise that where a true physical interaction occurs between proteins X and Y, then if protein X pulls down protein Y, protein Y should by corollary pull down protein X. The same APMS protocol used in the primary screen was employed (Supplemental Table S6). All three bait proteins were identified *via* multiple peptides by tandem mass spectrometry, with a minimum coverage of 28% amino acid sequence for P0A138 and a maximum of 55% for Q88F99. Additionally, all bait proteins were significantly enriched in the cognate APMS sample relative to null control (unfused eYFP), thereby confirming that the baits were successfully expressed and that the APMS experiment was effective (Fig. 4*A*).

PDH-E1 is a component of the pyruvate dehydrogenase complex, which catalyzes the conversion of pyruvate to acetyl-CoA and carbon dioxide. This large (>5 MDa) complex is composed of multiple copies of pyruvate dehydrogenase (E1), dihydrolipoamide acetyltransferase (E2), and lipoamide dehydrogenase (E3). We observed the E2 subunit in our APMS experiments but not the E3 subunit. Furthermore, both known phasins, PhaI and Pha F were significantly enriched in this pulldown. This suggests that PDH-E1 is a novel GAP.

OprL/Pal and its partner TolB (P0A173) were both identified,. While several other Tol proteins form parts of the Pal-Tol system, they are located in the inner membrane whereas TolB is located in the periplasm. This fact, combined with independent evidence that TolB interacts directly with OprL/Pal (33), suggests that the APMS experiment was successful. Furthermore, the phasin PhaF (but not PhaI) was significantly enriched in the pulldown. This strengthens the argument that OprL is a *bone fide* component of the carbonosome and a candidate new phasin, thereby establishing a physical link connecting PHA biosynthetic pathways and pyruvate metabolism.

Putative Lipoprotein (Q88F99): Q88F99 was selected as an exemplar lipoprotein. Four proteins were significantly enriched by Q88F99 pulldown. In addition to the bait, the chaperone DnaK, hydroxyproline acetylase, and N-succinyl-L,L-diaminopimelate aminotransferase, were identified as putative interactors. However, no known carbonosome or PHA-related proteins were identified, and it seems unlikely that Q88F99 is a true carbonosome protein.

### Localization Studies of Putative GAPs

Proteins that physically interact are likely to share the same subcellular location. We therefore used confocal microscopy to investigate the subcellular localization of potential interactors identified in our screens. Cultures expressing eYFP-tagged proteins of interest were grown in PHA accumulating conditions (N-limited), harvested after 24 h, and stained using Nile red. As expected, the fluorescent signal in cells expressing unfused eYFP (null control) was distributed throughout the cell, whereas the Nile red staining was restricted to the granules (Fig. 4*B*). eYPF fusions to PhaI and PhaC1 served as positive controls since both are reported to localize to the carbonosome (34).

In these experiments, we found that signal from PDH-E1-eYFP is concentrated in the carbonosomes. In combination with evidence from the proteomics experiments, this strongly argues that PDH-E2 is a true carbonosome component. Similarly, we confirmed that the fluorescence signal from Pal-eYFP overlapped with Nile red signal, indicating that it is also a newly identified GAP. In contrast, signal from Q88F99-eYFP (putative lipoprotein) was expressed throughout the cell, adding to the evidence that is not a true GAP.

### Analysis of Disrupted GAP Function

**Fig. 5:**
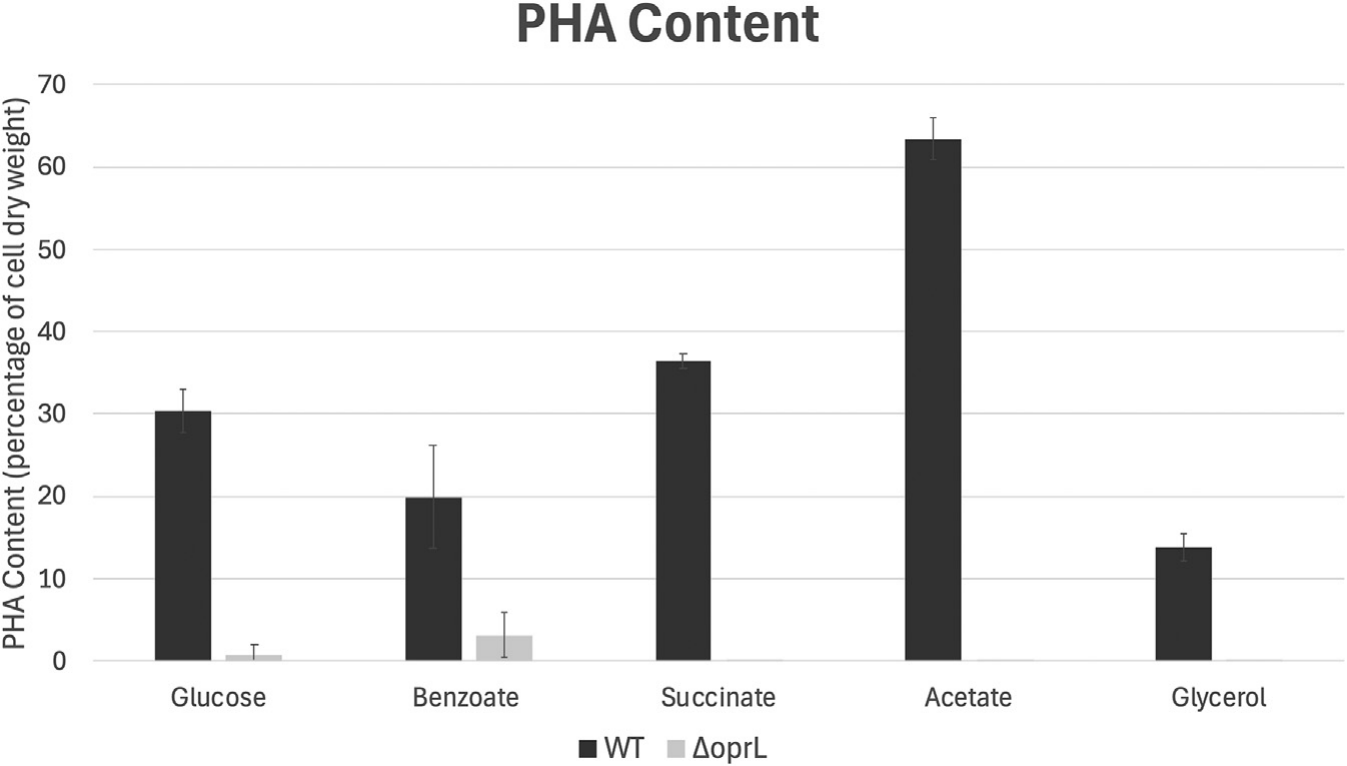
**Analysis of disrupted OprL function.** The percentage of PHA accumulated in KT2440 wild-type and ΔoprL grown in Minimal Salts media with nitrogen limitation is shown, determined by gas chromatography following acidic methanolysis of cells harvested after 48 h.

Finally, we investigated the PHA phenotype associated with the disruption of the *oprL* gene. A null mutant was generated using CRISPR reagents (Fig. 5*A*). The PHA content of the ΔoprL mutant was reduced compared to the wild type in cells grown in nitrogen-limited media (Fig. 5*B*). These experiments strongly suggest that OprL is located in the carbonosome and furthermore, that it plays a functional role in PHA biology.

## Discussion

Network approaches are optimal for studying systems containing many interacting components, where the analysis of one component at a time may only provide limited contextual information. Here we used three powerful proteomics approaches to investigate PHA biology in *P.putida*: global expression analysis; analysis of the carbonosome proteome; and protein interaction mapping centered on the PHA pathway. In particular, we aimed to provide a more comprehensive view of PHA pathway regulation under nitrogen stress. PHA content, size, and distribution of carbonosomes within growing and dividing cells are tightly controlled in response to nitrogen status. The transcription factor PhaD drives expression from the *phaC1ZC2D* and *phaIF* operons *via* the PC1 and PI promoters (35). However, the exact mechanism by which nitrogen limitation more generally induces PHA accumulation is not fully understood. In common with other PHA-producing microorganisms, nitrogen control in *P.putida* is governed by a mixture of pathways that include both specific and global regulation (reviewed in Prieto 2014; (36). Disruption of several global regulation factors causes PHA accumulation phenotypes including RpoN (37), RpoS (38), PsrA (39), RelA/SpoT (40), PtsP/PtsO (41), and Crc (5). Moreover, as well as nitrogen stress, PHA accumulation is also reported to be enhanced under conditions of excess carbon, or the limitation of other nutrients, including sulfur, oxygen, phosphorous, or magnesium (42).

Here, we first used global expression proteomics (label-free quantitation) to compare protein levels under nitrogen-limited and nitrogen-unlimited growth conditions. Earlier proteomics studies addressing PHA production in *P.putida* used 2-dimensional gel electrophoresis to identify differentially regulated proteins between PHA accumulating and non-accumulating conditions (7, 27, 28). Although 2D gel proteome analysis is widely employed in the proteomics field in bacteria, it captures only a relatively small portion of the entire proteome expressed under the tested conditions. For example, while important data describing metabolic pathways were obtained in these studies, the central PHA-related proteins encoded by the *pha* locus were not identified. To improve insight into what proteins were changing between nitrogen-limited and nitrogen-excess conditions, we applied sensitive LCMS techniques (high resolution, high mass accuracy, label-free quantitative proteomics) to the entire solubilized *P.putida* proteome. This approach was earlier implemented by Nikodinovic-Runic and coworkers [2009] to identify proteins in *P.putida* CA-3 grown on styrene. Nikodinovic-Runic observed PhaC1, PhaC2, PhaI, and PhaF expression under nitrogen-limiting conditions. These were among the >1800 proteins we quantified in a 48 h growth time course, providing an overview of the proteome-wide response to nitrogen stress and mapping to clustered pathways.

We next focused on the carbonosome, the site of PHA storage in *P.putida* cells. Fewer than 10 proteins have been confirmed by microscopy studies to localize to the carbonosome (34). However, many hundreds of proteins have been reported in studies describing carbonosome preparations. These high numbers may arise from the high sensitivity of modern mass spectrometry platforms used. Alternatively, they may reflect inefficient purification procedures, resulting in a large faction of nonspecific carry-over proteins (31). A third category of low stoichiometry interactions may represent low affinity or transient interactions, which nevertheless may mediate important functional roles in the carbonosome. We acknowledge that our stringent bioinformatic analysis precludes the identification of these proteins; however, all the data is available for scrutiny in Supplemental Table S4. Studies using the less sensitive MALDI approach typically identify approximately 10 to 20 abundant proteins that can be visualized and cut out from an SDS-PAGE gel. These proteins clearly are the important contributors to the protein component of the carbonosomes by mass; nevertheless, the use of LCMS to search for minor components that may have functional importance is justified, provided rigorous methods and controls are used to minimize the probability of false positives. One example of a protein that may be a non-specific binder is the outer membrane porin OrpF (Q88L46). This was ranked fourth most abundant protein in our carbonosome proteome analysis and we also found it to be upregulated in nitrogen-limited conditions. Moreover, Tarazona and coworkers [2020] identified OprF as both a carbonosome-associated protein and an interacting partner of PhaF. However, Tarazona did not find further evidence to support the interaction with PhaF. OprF is an OmpA family member and a major protein component of the outer membrane of the Pseudomonads. This protein has been extensively studied, particularly in *Pseudomonas aeruginosa* because of its role in antimicrobial drug resistance and porin function, and in the maintenance of cell shape and membrane integrity. It is possible that OprF opportunistically binds to PHA granules during cell lysis due to its abundance in the cell, as suggested by (31).

In a third proteomics strategy, the physical interactions of PHA-related proteins were mapped using an APMS approach in order to gain a better understanding of how they are integrated in the cellular network. Surprisingly, there was little overlap in potential interacting partners among PHA-related proteins. However, our work does support the interactions observed between phasins PhaI and PhaF by Tarazona and coworkers [2020]. One potential explanation for the apparently low levels of mutual interaction is that interactions between PHA-related proteins are transient and cannot be detected using conventional affinity purification. A cross-link APMS approach may be useful in this regard. Alternatively, it is possible that although PHA-related proteins are co-localized on the PHA granule, they may not physically interact. PHA synthesis and degradation is a complex multienzyme process that is regulated at transcriptional, translational, and metabolic levels (43). Components of the PHA metabolic pathway may be regulated independently of each other and may have no physiological reason to interact.

We found evidence that two new proteins may be considered true GAPs, or carbonosome-associated proteins. The PDH-E1 was identified in our N-limited enrichment proteome dataset as well as our carbonosome proteome. It was classed as a putative interactor of PhaI following the first round of PPI screen, and this observation was confirmed when the reverse orientation PPI experiment was carried out in round 2. Localization to the carbonosome was also confirmed using fluorescence microscopy. We conclude that PDE-E1 is a newly identified carbonosome component, and therefore physically linked to the PHA pathways in *P.putida*. The significance of this observation is unknown. However, the conversion of pyruvate to acetyl CoA by PDH is integral to PHA production, as it provides the carbon source for *de novo* fatty acid synthesis (44). In part support of this, overexpression of the pyruvate dehydrogenase E1 subunit in *P.putida* cells grown on glucose resulted in a 33% increase in accumulated PHA (45). Furthermore, acyl-CoA synthase (Acs1), which catalyzes the first step in the activation of fatty acids for the β-oxidation cycle, was previously shown to localize and have activity in carbonsome preparations from *P.putida* (46). This suggests that at least some of the biochemical activity associated with PHA metabolism may be functionally as well as physically located at the carbonosomes.

The peptidoglycan-associated lipoprotein OprL/Pal was upregulated following growth in N-limited media. It was also found in carbonosome purifications in the current manuscript and by (13). We confirmed this using fluorescence microscopy localization experiments. OprL was also found to interact with PhaI in our first PPI screen, and we confirmed the interaction in a second screen where PhaI was in turn co-purified in an OprL pulldown experiment. The significance of the interaction is unclear. OprL/Pal forms a large hetero-oligocomplex with other members of the Pal-Tol system which creates a network linking the inner and outer bacterial membranes and the peptidoglycan layer. The complex is vital for maintaining the integrity of the membrane and it is possible that it is involved in regulating or affecting the distribution of carbonosomes to daughter cells during cell division. In fact, a role for the Pal-Tor complex in bacterial cell division was reported recently (47). In further support of this, a mutant was recently identified in a transposon screen for disrupted PHB (poly-β-hydroxybutyrate) accumulation in *Azotobacter vinelandii* that mapped to the homolog of the *oprL* gene (48). This mutant showed an abnormal distribution of proteins on isolated carbonosomes. To investigate further, we disrupted oprL function, finding a similar effect on PHA accumulation in *P.putida* KT2440.

## Data Availability

Raw data, including results files and software needed for viewing spectra is deposited in the ProteomeXchange Consortium *via* the PRIDE partner repository with the dataset identifier PXD045993 (49).

## Supplemental data

This article contains supplemental data.

## Conflict of interest

The authors declare that they have no known competing financial interests or personal relationships that could have appeared to influence the work reported in this paper.

## References

[1] Bhola S., Arora K., Kulshrestha S., Mehariya S., Bhatia R.K., Kaur P. (2021). Established and emerging producers of PHA: redefining the possibility. Appl. Biochem. Biotechnol..

[2] Geyer R., Jambeck J.R., Law K.L. (2017). Production, use, and fate of all plastics ever made. Sci. Adv..

[3] Singh A.K., Srivastava J.K., Chandel A.K., Sharma L., Mallick N., Singh S.P. (2019). Biomedical applications of microbially engineered polyhydroxyalkanoates: an insight into recent advances, bottlenecks, and solutions. Appl. Microbiol. Biotechnol..

[4] Dabrowska D., Mozejko-Ciesielska J., Pokój T., Ciesielski S. (2020). Transcriptome changes in Pseudomonas putida KT2440 during medium-chain-length polyhydroxyalkanoate synthesis induced by nitrogen limitation. Int. J. Mol. Sci..

[5] La Rosa R., de la Peña F., Prieto M.A., Rojo F. (2014). The Crc protein inhibits the production of polyhydroxyalkanoates in Pseudomonas putida under balanced carbon/nitrogen growth conditions. Environ. Microbiol..

[6] Nikodinovic-Runic J., Flanagan M., Hume A.R., Cagney G., O'Connor K.E. (2009). Analysis of the Pseudomonas putida CA-3 proteome during growth on styrene under nitrogen-limiting and non-limiting conditions. Microbiology (Reading).

[7] Możejko-Ciesielska J., Serafim L.S. (2019). Proteomic response of Pseudomonas putida KT2440 to dual carbon-Phosphorus limitation during mcl-PHAs synthesis. Biomolecules.

[8] Escapa I.F., García J.L., Bühler B., Blank L.M., Prieto M.A. (2012). The polyhydroxyalkanoate metabolism controls carbon and energy spillage in Pseudomonas putida. Environ. Microbiol..

[9] Tribelli P.M., Pezzoni M., Brito M.G., Montesinos N.V., Costa C.S., López N.I. (2020). Response to lethal UVA radiation in the Antarctic bacterium Pseudomonas extremaustralis: polyhydroxybutyrate and cold adaptation as protective factors. Extremophiles.

[10] Obruca S., Sedlacek P., Mravec F., Krzyzanek V., Nebesarova J., Samek O. (2017). The presence of PHB granules in cytoplasm protects non-halophilic bacterial cells against the harmful impact of hypertonic environments. N. Biotechnol..

[11] Obruca S., Sedlacek P., Krzyzanek V., Mravec F., Hrubanova K., Samek O. (2016). Accumulation of poly(3-hydroxybutyrate) helps bacterial cells to Survive Freezing. PLoS One.

[12] Mezzina M.P., Pettinari M.J. (2016). Phasins, Multifaceted polyhydroxyalkanoate granule-associated proteins. Appl. Environ. Microbiol..

[13] Tarazona N.A., Hernández-Arriaga A.M., Kniewel R., Prieto M.A. (2020). Phasin interactome reveals the interplay of PhaF with the polyhydroxyalkanoate transcriptional regulatory protein PhaD in Pseudomonas putida. Environ. Microbiol..

[14] Galán B., Dinjaski N., Maestro B., de Eugenio L.I., Escapa I.F., Sanz J.M. (2011). Nucleoid-associated PhaF phasin drives intracellular location and segregation of polyhydroxyalkanoate granules in Pseudomonas putida KT2442. Mol. Microbiol..

[15] Lageveen R.G., Huisman G.W., Preusting H., Ketelaar P., Eggink G., Witholt B. (1988). Formation of Polyesters by Pseudomonas oleovorans: effect of substrates on Formation and composition of poly-(R)-3-Hydroxyalkanoates and poly-(R)-3-Hydroxyalkenoates. Appl. Environ. Microbiol..

[16] Ansari N.F., Amirul A.A. (2013). Preparation and characterization of polyhydroxyalkanoates macroporous scaffold through enzyme-mediated modifications. Appl. Biochem. Biotechnol..

[17] Wiśniewski J.R., Zougman A., Nagaraj N., Mann M. (2009). Universal sample preparation method for proteome analysis. Nat. Methods.

[18] Tyanova S., Temu T., Cox J. (2016). The MaxQuant computational platform for mass spectrometry-based shotgun proteomics. Nat. Protoc..

[19] Tyanova S., Temu T., Sinitcyn P., Carlson A., Hein M.Y., Geiger T. (2016). The Perseus computational platform for comprehensive analysis of (prote)omics data. Nat. Methods.

[20] Cook T.B., Rand J.M., Nurani W., Courtney D.K., Liu S.A., Pfleger B.F. (2018). Genetic tools for reliable gene expression and recombineering in Pseudomonas putida. J. Ind. Microbiol. Biotechnol..

[21] Shannon P., Markiel A., Ozier O., Baliga N.S., Wang J.T., Ramage D. (2003). Cytoscape: a software environment for integrated models of biomolecular interaction networks. Genome Res..

[22] Bindea G., Mlecnik B., Hackl H., Charoentong P., Tosolini M., Kirilovsky A. (2009). ClueGO: a Cytoscape plug-in to decipher functionally grouped gene ontology and pathway annotation networks. Bioinformatics.

[23] Mészáros B., Erdos G., Dosztányi Z. (2018). IUPred2A: context-dependent prediction of protein disorder as a function of redox state and protein binding. Nucleic Acids Res..

[24] Almagro Armenteros J.J., Tsirigos K.D., Sønderby C.K., Petersen T.N., Winther O., Brunak S. (2019). SignalP 5.0 improves signal peptide predictions using deep neural networks. Nat. Biotechnol..

[25] Sonnhammer E.L., von Heijne G., Krogh A. (1998). A hidden Markov model for predicting transmembrane helices in protein sequences. Proc. Int. Conf. Intell. Syst. Mol. Biol..

[26] Krug K., Carpy A., Behrends G., Matic K., Soares N.C., Macek B. (2013). Deep coverage of the Escherichia coli proteome enables the assessment of false discovery rates in simple proteogenomic experiments. Mol. Cell. Proteomics.

[27] Poblete-Castro I., Escapa I.F., Jäger C., Puchalka J., Lam C.M., Schomburg D. (2012). The metabolic response of P. putida KT2442 producing high levels of polyhydroxyalkanoate under single- and multiple-nutrient-limited growth: highlights from a multi-level omics approach. Microb. Cell Fact..

[28] Możejko-Ciesielska J., Mostek A. (2019). A 2D-DIGE-based proteomic analysis brings new insights into cellular responses of Pseudomonas putida KT2440 during polyhydroxyalkanoates synthesis. Microb. Cell Fact..

[29] Catone M.V., Ruiz J.A., Castellanos M., Segura D., Espin G., López N.I. (2014). High polyhydroxybutyrate production in Pseudomonas extremaustralis is associated with differential expression of horizontally acquired and core genome polyhydroxyalkanoate synthase genes. PLoS One.

[30] Tirapelle E.F., Müller-Santos M., Tadra-Sfeir M.Z., Kadowaki M.A., Steffens M.B., Monteiro R.A. (2013). Identification of proteins associated with polyhydroxybutyrate granules from Herbaspirillum seropedicae SmR1--old partners, new players. PLoS One.

[31] Jendrossek D., Pfeiffer D. (2014). New insights in the formation of polyhydroxyalkanoate granules (carbonosomes) and novel functions of poly(3-hydroxybutyrate). Environ. Microbiol..

[32] Maestro B., Galán B., Alfonso C., Rivas G., Prieto M.A., Sanz J.M. (2013). A new family of intrinsically disordered proteins: structural characterization of the major phasin PhaF from Pseudomonas putida KT2440. PLoS One.

[33] Szczepaniak J., Press C., Kleanthous C. (2020). The multifarious roles of Tol-Pal in Gram-negative bacteria. FEMS Microbiol. Rev..

[34] Prieto A., Escapa I.F., Martínez V., Dinjaski N., Herencias C., de la Peña F. (2016). A holistic view of polyhydroxyalkanoate metabolism in Pseudomonas putida. Environ. Microbiol..

[35] de Eugenio L.I., Galán B., Escapa I.F., Maestro B., Sanz J.M., García J.L. (2010). The PhaD regulator controls the simultaneous expression of the pha genes involved in polyhydroxyalkanoate metabolism and turnover in Pseudomonas putida KT2442. Environ. Microbiol..

[36] Mitra R., Xu T., Chen G.Q., Xiang H., Han J. (2022). An updated overview on the regulatory circuits of polyhydroxyalkanoates synthesis. Microb. Biotechnol..

[37] Hoffmann N., Rehm B.H. (2005). Nitrogen-dependent regulation of medium-chain length polyhydroxyalkanoate biosynthesis genes in pseudomonads. Biotechnol. Lett..

[38] Raiger-Iustman L.J., Ruiz J.A. (2008). The alternative sigma factor, sigmaS, affects polyhydroxyalkanoate metabolism in Pseudomonas putida. FEMS Microbiol. Lett..

[39] Fonseca P., de la Peña F., Prieto M.A. (2014). A role for the regulator PsrA in the polyhydroxyalkanoate metabolism of Pseudomonas putida KT2440. Int. J. Biol. Macromol..

[40] Mozejko-Ciesielska J., Dabrowska D., Szalewska-Palasz A., Ciesielski S. (2017). Medium-chain-length polyhydroxyalkanoates synthesis by Pseudomonas putida KT2440 relA/spoT mutant: bioprocess characterization and transcriptome analysis. AMB Express.

[41] Velázquez F., Pflüger K., Cases I., De Eugenio L.I., de Lorenzo V. (2007). The phosphotransferase system formed by PtsP, PtsO, and PtsN proteins controls production of polyhydroxyalkanoates in Pseudomonas putida. J. Bacteriol..

[42] Madkour M.H., Heinrich D., Alghamdi M.A., Shabbaj I.I., Steinbüchel A. (2013). PHA recovery from biomass. Biomacromolecules.

[43] López N.I., Pettinari M.J., Nikel P.I., Méndez B.S. (2015). Polyhydroxyalkanoates: Much more than biodegradable plastics. Adv. Appl. Microbiol..

[44] Rehm B.H., Krüger N., Steinbüchel A. (1998). A new metabolic link between fatty acid de novo synthesis and polyhydroxyalkanoic acid synthesis. The PHAG gene from Pseudomonas putida KT2440 encodes a 3-hydroxyacyl-acyl carrier protein-coenzyme a transferase. J. Biol. Chem..

[45] Borrero-de Acuña J.M., Bielecka A., Häussler S., Schobert M., Jahn M., Wittmann C. (2014). Production of medium chain length polyhydroxyalkanoate in metabolic flux optimized Pseudomonas putida. Microb. Cell Fact..

[46] Ruth K., de Roo G., Egli T., Ren Q. (2008). Identification of two acyl-CoA synthetases from Pseudomonas putida GPo1: one is located at the surface of polyhydroxyalkanoates granules. Biomacromolecules.

[47] Yakhnina A.A., Bernhardt T.G. (2020). The Tol-Pal system is required for peptidoglycan-cleaving enzymes to complete bacterial cell division. Proc. Natl. Acad. Sci. U. S. A..

[48] Moreno S., Castellanos M., Bedoya-Pérez L.P., Canales-Herrerías P., Espín G., Muriel-Millán L.F. (2019). Outer membrane protein I is associated with poly-β-hydroxybutyrate granules and is necessary for optimal polymer accumulation in Azotobacter vinelandii on solid medium. Microbiology (Reading).

[49] Vizcaíno J.A., Csordas A., Del-Toro N., Dianes J.A., Griss J., Lavidas I. (2016). 2016 update of the PRIDE database and its related tools. Nucleic Acids Res..

[50] Obradovic Z., Peng K., Vucetic S., Radivojac P., Brown C.J., Dunker A.K. (2003). Predicting intrinsic disorder from amino acid sequence. Proteins.

